# Optimal Extraction and Deproteinization Method for Mannoprotein Purification from *Kluyveromyces marxianus*

**DOI:** 10.61186/ibj.27.5.320

**Published:** 2023-02-12

**Authors:** Ashraf Hajhosseini, Anousheh Sharifan, Zohre Eftekhari, Ariana Alavi, Delaram Doroud

**Affiliations:** 1 Department of Food Science and Technology, Science and Research Branch, Islamic Azad University, Tehran, Iran;; 2 Biotechnology Department, Pasteur Institute of Iran, Tehran, Iran;; 3 Production and Research Complex, Pasteur Institute of Iran, Tehran, Iran

**Keywords:** Glycoproteins, Kluyveromyces marxianus, Mannans

## Abstract

**Background:**

Mannoproteins, mannose-glycosylated proteins, play an important role in biological processes and have various applications in industries. Several methods have been already used for the extraction of mannoproteins from yeast cell-wall. The aim of this study was to evaluate the extraction and deproteinization of mannan oligosaccharide from the *K. marxianus* mannoprotein.

**Methods:**

To acquire crude mannan oligosaccharides, *K. marxianus *mannoproteins were deproteinized by the Sevage, TCA, and HCL methods. Total nitrogen, crude protein content, fat, carbohydrate and ash content were measured according to the monograph prepared by the meeting of the Joint FAO/WHO Expert Committee and standard. Mannan oligosaccharide loss, percentage of deproteinization, and chemical composition of the product were assessed to check the proficiency of different methods.

**Results:**

Highly purified (95.4%) mannan oligosaccharide with the highest deproteinization (97.33 ± 0.4%) and mannan oligosaccharide loss (25.1 ± 0.6%) were obtained following HCl method.

**Conclusion:**

HCl was the most appropriate deproteinization method for the removal of impurities. This preliminary data will support future studies to design scale-up procedures.

## INTRODUCTION

Yeast cell wall primarily consists of polymers of *β*-glucose, namely β-glucans, and manno-proteins. Most of the yeast cell wall proteins are linked to mannan oligosaccharides to make the mannoprotein complex. Therefore, mannoproteins with a highly glycosylated structure lie in the outer layer of yeast cells and are considered structural components^[^^1^^]^. Due to the molecular and structural characteristics, mannoproteins have found many techno-functional applications, particularly in food industry^[^^2^^]^. For instance, in salad dressing or mayonnaise, mannoproteins can act as a natural emulsifier and stabilizer with no undesirable effect on the sensory properties of the final products^[^^3^^,^^4^^]^. Besides these features, mannoproteins are of great importance for human health given their growth-promoting impact on lactic acid bacteria^[^^5^^,^^6^^]^ and macrophage^[^^7^^-^^9^^]^, as well as for their inhibitory actions on pathogenic bacteria^[^^10^^]^, free radicals, and cancer cells^[^^7^^,^^8^^]^. It has been reported that these beneficial effects may vary depending on mannoprotein-related factors, such as molecular weight and monosaccharide composition^[^^11^^]^. 

Mannan is a highly branched polysaccharide linked to proteins and has a backbone of α-(1→6)-linked mannose units with α-(1→2, 3)-linked side chains (also known as mannan oligosaccharides). Of these biomolecules, mannan oligosaccharides, as an indigestible short-chain polymeric material, is a prominent supplement enables the improvement of growth performance and body weight in many animals^[^^12^^,^^13^^]^. Also, it is effective in suppressing the onset of atherosclerosis development via the reduction of plasma cholesterol levels^[^^14^^]^. In common with mannan, β-glucans are conserved structural components in the yeast cell walls and act as biological response modifiers in promoting the immune system^[^^15^^]^. These natural materials in the form of microparticles can be used not only as immunostimulants but also as antigen carriers. They contribute, for the most part, to mucosal vaccination^[^^16^^]^. This yeast cell wall is made of 30-60% polysaccharides (β-glucan and mannan oligo-saccharides), 15-30% proteins, 5-20% lipids, and a small amount of chitin. In terms of the structure, mannan exists in different forms, such as the linear mannan, glucomannan (mannose and glucose in 3:1 ratio), galactomannan (mannose, glucose, and galactose in 3:1:1 ratio), and galactoglucomannan^[^^11^^]^. Mannan, harboring mannose polymers and the protein bounds, shows the structure of a natural amphiphilic biopolymer compound. 

The present study focused on mannoprotein and mannan oligosaccharide, which its supply appears to be insufficient for meeting the current and future demands^[^^17^^]^. Therefore, for the production of mannan oligosaccharide, an efficient and economically viable method needs to be investigated and developed. So far, the biotechnological properties of the yeast mannoprotein are still an attractive field of research. *K. marxianus*, a Crabtree-negative yeast, has the potential to produce valuable chemicals. It is able to grow quickly, utilize many sugars, and produce a large number of proteins^[^^18^^,^^19^^]^. These reasons persuaded us to consider this prospective micro-organism in this study to evaluate the extraction and deproteinization of mannan oligosaccharide from the *K. marxianus *mannoprotein. Several methods are used for the isolation and purification of mannoproteins from yeast cell wall. Amongst them, three methods of extraction and deproteinization, including Sevage, TCA, and HCl, were examined. These approaches were chosen according to their cost-effectiveness and scale-up ability while considering the possibility of implementing in an industrial facility. 

## MATERIALS AND METHODS

All the chemicals used in the study were of analytical grade and supplied by Merck, Germany. Yeast strain and inoculum stock preparation, *K. marxianus* (IBRC-M no. 30114), was obtained from the Iranian Biological Resource Center, Tehran, Iran. After activation, the cells were kept on yeast-peptone-dextrose agar slants at 4 °C for 12 h. To prepare 100 mL of the culture medium, peptone (2 g), glucose (2 g), and yeast extract (1 g) were mixed with double distillation water. 

**Inoculum development**

A loop of the yeast colonies was transferred to 100 mL of inoculum medium and subsequently placed in a shaking incubator (IKA, Germany) at 180 rpm at 28 °C for 24 h^[^^20^^-^^22^^]^.

**Isolation and preparation of the yeast cells**

The yeast cells were collected by the centrifugation of the culture medium at 2000 ×g for 10 min. After rinsing with cool deionized water, the cells were mixed with glass beads and agitated rigorously using a vortex mixer while keeping on ice. After removing the glass beads, the resultant suspension was underwent centrifugation at 2000 ×g for 10 min. The precipitated cells were then rinsed several times with cool deionized water^[^^22^^-^^24^^]^. 

**Extraction and purification of the mannoprotein**

The yeast cell precipitate acquired from the previous step was resuspended in 20% w/w buffer solution (0.1 M of potassium citrate and 0.02 M of sodium metabisulfite at pH 7.0) and kept at 121 °C and 1.2 bar for 90 min^[^^25^^]^. The mannoprotein was washed with acetic acid (1%) in ethanol (96%) after being precipitated by centrifugation at 700 ×g for 10 min. The supernatant was maintained in incubated at 4 °C overnight and then centrifuged at 5000 ×g for 10 min to complete the precipitation^[^^4^^,^^23^^]^. Hexadecyltrimethyl-ammonium bromide, with the chemical formula C_16_H_33_N(CH_3_)_3_ Br (Merck), was used as a solvent for the selective precipitation and purification of the mannoprotein from other macromolecules, such as proteins. The obtained precipitate was underwent dialysis against deionized water for 48 h to finalize the purification process^[^^22^^]^.

**Biomass quantity measurement**

The yeast cells (yeast suspension) were isolated by applying centrifugation at 2000 ×g for 10 min. The cells were then collected and dried in an oven at 105 °C for 5 h to reach a constant weight.

**Extraction of the crude mannan oligosaccharides**

 The water-soluble mannan oligosaccharides were extracted from 5 g of the yeast cell walls in 1% NaOH (50 mL) at 100 °C for 2 h before cooling and neutralizing to pH 7 with dilute HCl solution. After filtration, the mannan oligosaccharides were precipitated by adding 200 mL of absolute ethanol. Using absolute ethanol and diethyl ether, the precipitate was washed^[^^23^^,^^26^^,^^27^^]^.

**Deproteinization by the Sevage, TCA, and HCL methods**

Deproteinization was carried out using Sevage, TCA, and HCl. In the Sevage method, the concentrated solution of the crude mannan oligosaccharides was combined with 0.2 volumes of chloroform/isoamyl alcohol (5:1 v/v) and strongly shaken in a separatory funnel for 5 min. The aqueous phase was centrifuged at 500 ×g at 2-5º C for 10 min, and the aqueous layer was drawn off from the remaining chloroform layer cautiously. For three to five times, this process was repeated until no further precipitate was seen at the interface. The mannan oligosaccharides were subsequently precipitated from the aqueous phase with three to four volumes of ethanol^[^^27^^,^^28^^]^. In TCA and HCL methods, the concentrated solution of the crude mannan oligosaccharides was adjusted to pH 3 with 10% TCA solution and HCL (2M). The sample was centrifuged at 2800 ×g for 10 min at 2-5º C, and the precipitate was discarded to obtain the deproteinized solution. 

**Chemical analyses**

A Kjeldahl analyzer (Foss-2300 Kjeltec, Sweden) was employed to measure total nitrogen. The crude protein content was computed by multiplying the amount of total nitrogen by 6.25. The soluble protein content was determined according to a previously described method^[^^29^^]^. Official Methods of Analysis of Association of Official Analytical Chemists (AOAC) were utilized to determine total fat, as well as ash content^[^^30^^,^^31^^]^. Considering petroleum ether as the organic solvent, the fat determination was conducted by a Soxhlet extraction apparatus (Foss-2050 Soxtec, Sweden). Ash content was determined by incinerating dried samples in a furnace (Lindberg/Blue M, USA) at 600 °C for about 5 h. The amount of total carbohydrate was determined by the use of the phenol-sulfuric acid method^[^^31^^]^.

**Statistical analysis**

Data in this study were processed by applying the SPSS Statistics version 20.0. The results were described as mean values ± standard deviation of at least four replicate experiments. The one-way ANOVA test and Duncan’s multiple range tests were applied at a statistically significant level of less than 0.05 to evaluate the differences.

## RESULTS AND DISCUSSION

*K. marxianus *is a yeast species with various industrial applications. Mannoprotein, a mannose glycosylated protein, is found in the cell wall of this yeast and plays important roles in various biological processes with several applications in different industries. The protein part of this molecule may interact with other molecules and have an impact on cellular behavior, especially in the field of immunology science^[^^32^^,^^33^^]^. According to the important biological functions of crude mannan oligo-saccharides, several methods of its deproteinization have been studied to validate an appropriate downstream process to obtain pure mannan oligo-saccharides. In this regard, three methods, including the Sevage, TCA, and HCL were investigated. The principle of the Sevage method is denaturating of the dissociative protein by an organic solvent while in the TCA method, the cationic protein binds to the TCA to be precipitated. Moreover, in the hydrochloric acid method, the deproteinization will occur because the solubility of protein in the crude mannoprotein is the lowest at isoelectric point. 

The results of deproteinization are shown in Table 1. Overall, the HCl method had the highest percentages of deproteinization and mannan oligosaccharide loss. Statistical analysis exhibited no significant difference in deproteinization after applying these methods (p > 0.05). While the percentage of mannan oligosaccharide loss in HCl almost doubled that of the Sevage method, mannan oligosaccharide loss did not vary significantly among these methods (p > 0.05). Indeed, Sevage reagent and TCA cause proteins to be denatured; therefore, centrifugation is used to completely remove the remaining protein. During the Sevage method, no damage occurred in the polysaccharide structure due to the mild conditions of the procedure; however, the process was repeated several times to compensate the poor deproteinization effect. There are some reports demonstrating that TCA method induces unwanted destructive impact on the polysaccharide structure^[^^34^^]^. Thus, it seems that the percentage of mannan oligosaccharide loss in the process of extraction utilizing the TCA method is higher than the Sevage method (16.6 ± 1.30% vs. 12.5 ± 0.8%). Duan *et al.*^[^^34^^]^ reported higher deproteinization efficiency and polysaccharide recovery ratio utilizing the Sevage extraction method for rapeseed meal polysaccharides. Furthermore, the percentages of deproteinization and mannan oligosaccharide loss in the HCl extraction method was higher than the TCA extraction method. Huang *et al.*^[^^35^^]^ also reported higher percentages of deproteinization and polysaccharide loss (98.6% and 29%, respectively) in the crude cell suspension of garlic. In another study on the extraction of mannan oligosaccharides from the yeast cell wall, Huang *et al.*^[^^36^^]^ showed high efficiency of the HCl method in reporting deproteinization percentage (96.7%), as compared to the Sevage (89.8%) and TCA (91.4%) methods^[^^37^^]^. In addition to the low ability to deproteinize crude extracts, the Sevage method utilizes organic solvents, e.g. chloroform, which is a poisonous non environmental friendly solvent^[^^36^^]^. 

**Table 1:** Comparison of three deproteinization methods

**Method**	**Deproteinization (%)**	**Mannan oligosaccharide loss (%)**
Sevage	87.50 ± 0.5	12.5 ± 0.8
TCA	90.64 ± 0.7	16.6 ± 1.3
HCl	97.33 ± 0.4	25.1 ± 0.6

Data were presented by the mean ± standard deviation.

**Table 2:** Compositions of yeast, its cell wall, crud mannoprotein, and purified mannan oligosaccharides

**Content**	***K. marxianus***					
	**Yeast (%)**	**Yeast cell wall (%)**	**Crude mannoprotein** **(%)**	**Purified mannan oligosaccharides (%)**		
				**Sevage ** **method**	**TCA ** **method**	**HCl ** **method**
Dry weight	100	26.08 ± 0.4	8.42 ± 0.06	7.03 ± 0.25	6.91 ± 0.15	6.84 ± 0.3
Carbohydrate (as glucose)	39.09 ± 1.07	88.3 ± 0.66	71.25 ± 0.37	89.11 ± 1.2	84.37 ± 1.28	78.92 ± 1.01
Mannan	11.26 ± 0.41	38.02 ± 1.33	67.4 ± 1.3	86.62 ± 0.5	82.62 ± 0.43	75.01 ± 0.48
Protein	51.06 ± 3.11	10.24 ± 0.27	21.05 ± 0.45	11.88 ± 0.6^*^	8.04 ± 0.51^*^	3.46 ± 0.55^**^
Fat	4.55 ± 0.12	3.32 ± 0.18	Trace	Trace	Trace	Trace
Ash	6.9 ± 0.7	3.69 ± 0.2	2.34 ± 0.1	2.63 ± 0.2	1.85 ± 1.4	1.97 ± 0.86

All data represent dry weight ratios. Statistically significant differences are indicated by One-way ANOVA (^*^p < 0.05 and ^**^p < 0.001).

As summarized in Table 2, 26.08 ± 0.4% of the dry weight of the yeast cells are composed of the cell-wall components and 8.42 ± 0.06% mannoproteins. Also, 38.02 ± 1.33% of the yeast cell wall and 67.4 ± 1.3% of the crude mannoproteins are from Mannan oligosaccharide. This amount of mannan is 11.26 ± 0.41% of total yeast dry weight. It has also been documented that the yeast cell wall constitutes around 20% of the yeast cell dry weight^[^^1^^]^. Comparatively, the dry weight and extracted mannan content of the crude mannoprotein were similar to those reported by Liu *et al.*^[^^28^^]^. When the Sevage method was implemented for the extraction of the mannan oligosaccharide, the amount of the total recovered carbohydrate was 89.11 ± 1.2% (86.62 ± 0.5% as mannan), with 11.88 ± 0.6% protein content and 2.63 ± 0.2% remained ash. It is worth to mention that the use of different deproteinization methods had no significant effect on chemical composition. Following the HCl extraction method, the mannan polysaccharide with the significantly higher purity with produced based on the percent protein content (3.46 ± 0.55%) as compared to the other methods (p < 0.001). Regardless of the deproteinization methods, carbohydrate portion was the foremost component of the purified mannan oligosaccharide, as expected. The efficiency of Sevage method for the removal of protein impurity was significantly lower than the other methods. It seems that the advantage of the mentioned method was its remarkable ability to extract a great amount of carbohydrates from the cell wall of the yeast. Despite this advantage, the ability of this method to decrease the protein as an impurity was unsatisfactory compared to the other methods.

In this study, three different deproteinization methods were utilized to purify mannan oligosaccharide from *K. marxianus *mannoproteins. The highest deproteinization (97.33 ± 0.4%) and mannan oligosaccharide loss (25.1 ± 0.6%) were obtained when utilizing the HCl method. The mannan oligosaccharide processed by this method had the highest purity (95.4%), due to the remained protein as the impurity of the mannan bulk product. 

### Conclusion

Taken together, HCl extraction method for the purification of the mannan oligosaccharide has the advantage to efficiently remove the proteins from mannoprotein bulk in the yeast cell wall. The presence of the protein impurities makes a concern of interfering with the production of antigen-specific antibodies, while this oligosaccharide is used as an adjuvant as well as of interaction between mannan and the mannose receptor in the in vitro assays. Hence, the concept of removing of these proteins would be significantly a key process. Despite the higher percentage of mannan oligo-saccharide loss in the HCl method, it seems to be the most appropriate deproteinization method, in the terms of ability to remove the impurities while not using organic solvents that are toxic for the environment. This preliminary data will support future studies and verifications, for the design of scale-up procedures. 

## DECLARATIONS

### Acknowledgments

The present study was extracted from a Ph.D. thesis written by Ashraf Hajhosseini.

### Ethical statement

Not applicable.

### Data availability

The raw data supporting the conclusions of this article are available from the authors upon reasonable request.

### Author contributions

AH: performed the investigation, obtained the experimental data and wrote the manuscript; AS: contributed to analysis and verification of the data, advised scientifically and revised the manuscript, ZE: contributed to discuss the results and revised the manuscript, AA: performed the statistical analysis of the data, revised and wrote the manuscript; DD: conceptualized and designed the study, advised scientifically, verified the results, revised the manuscript critically

### Conflicts of interest:

None declared.

### Funding/support

The present study was financially supported by Pasteur Institute of Iran, Tehran (no. 1814).

## References

[1] Quirós M, Gonzalez R, Morales P (2012). A simple method for total quantification of mannoprotein content in real wine samples. Food chemistry.

[2] Caridi A (2006). Enological functions of parietal yeast mannoproteins. Antonie van leeuwenhoek.

[3] de Melo ANF, de Souza EL, da Silva Araujo VB, Magnani M (2015). Stability, nutritional and sensory characteristics of French salad dressing made with mannoprotein from spent brewer's yeast. LWT-Food science and technology.

[4] da Silva Araújo VB, de Melo ANF, Costa AG, Castro-Gomez RH, Madruga MS, de Souza EL, Magnani M (2014). Followed extraction of β-glucan and mannoprotein from spent brewer's yeast (Saccharomyces uvarum) and application of the obtained mannoprotein as a stabilizer in mayonnaise. Innovative food science and emerging technologies.

[5] Benyacoub J, Maulden GLC, Cavadini C, Sauthier T, Anderson RE, Schiffrin EJ, Weid TVD (2003). Supplementation of food with Enterococcis faecium (SF68) stimulates immune functions in young dogs. Journal of nutrition.

[6] Ganan M, Carrascosa AV, de Pascual Teresa S, Martinez Rodriguez AJ (2012). Effect of mannoproteins on the growth, gastrointestinal viability, and adherence to Caco‐2 cells of lactic acid bacteria. Journal of food science.

[7] Hosseini M, Sharifan A (2021). Biological properties of yeast-based mannoprotein for prospective biomedical applications. Combinatorial chemistry and high throughput screen.

[8] Roohvand F, Shokri M, Abdollahpour-Alitappeh M, Ehsani P (2017). Biomedical applications of yeast-a patent view, part one: yeasts as workhorses for the production of therapeutics and vaccines. Expert opinion on therapeutic patents.

[9] Darpossolo FPB, Eto SF, Venancio EJ, Castro Goméz RJH (2012). Saccharomyces uvarum mannoproteins stimulate a humoral immune response in mice. Brazilian archives of biology and technology.

[10] Ganan M, Carrascosa AV, de Pascual Teresa S, Martínez Rodríguez AJ (2009). Inhibition by yeast-derived mannoproteins of adherence to and invasion of Caco-2 cells by Campylobacter jejuni. Journal of food protection.

[11] Li J, Karboune S (2018). A comparative study for the isolation and characterization of mannoproteins from Saccharomyces cerevisiae yeast cell wall. International journal of biological macromolecules.

[12] Razeghi Mansour M, Akrami R, Ghobadi SH, Amani Denji K, Ezatrahimi N, Gharaei A (2012). Effect of dietary mannan oligosaccharide (MOS) on growth performance, survival, body composition, and some hematological parameters in giant sturgeon juvenile (Huso huso Linnaeus, 1754). Fish physiology and biochemistry.

[13] Dimitroglou A, Merrifield DL, Spring P, Sweetman J, Moate R, Davies SJ (2010). Effects of mannan oligosaccharide (MOS) supplementation on growth performance, feed utilisation, intestinal histology and gut microbiota of gilthead sea bream (Sparus aurata). Aquaculture.

[14] Hoving LR, van der Zande HJP, Pronk A, Guigas B, Willems van Dijk K, van Harmelen V (2018). Dietary yeast-derived mannan oligosaccharides have immune-modulatory properties but do not improve high fat diet-induced obesity and glucose intolerance. Plos one.

[15] Lew DB, LeMessurier KS, Palipane M, Lin Y, Samarasinghe AE (2018). Saccharomyces cerevisiae-Derived Mannan Does Not Alter Immune Responses to Aspergillus Allergens. Biomedical research international.

[16] Baert K, De Geest BG, De Greve H, Cox E, Devriendt B (2016). Duality of β-glucan microparticles: antigen carrier and immunostimulants. International journal of nanomedicine.

[17] Zhou M, Tao Y, Lai C, Huang C, Zhou Y, Yong Q (2019). Effects of mannanoligosaccharide supplementation on the growth performance, immunity, and oxidative status of partridge shank chickens. Animals (Basel).

[18] Fonseca GG, Heinzle E, Wittmann C, Gombert AK (2008). The yeast Kluyveromyces marxianus and its biotechnological potential. Applied microbiology and biotechnology.

[19] Lane MM, Morrissey JP (2010). Kluyveromyces marxianus: a yeast emerging from its sister's shadow. Fungal biology reviews.

[20] Aktaş N, Boyacı İH, Mutlu M, Tanyolaç A (2006). Optimization of lactose utilization in deproteinated whey by Kluyveromyces marxianus using response surface methodology (RSM). Bioresource technology.

[21] Eluk D, Ceruti R, Nagel O, Althaus R (2019). Effect of thermal treatment of whey contaminated with antibiotics on the growth of Kluyveromyces marxianus. Journal of dairy research.

[22] Hajhosseini A, Doroud D, Sharifan A, Eftekhari Z (2020). Optimizing growth conditions of Kluyveromyces marxianus for mannan production as a bioemulsifier. Applied food biotechnology.

[23] Hanneh VA, Ali MS, Elnaz M, Arash K, Masoome M (2014). Statistical optimization of culture media and conditions for maximize production of mannan by saccharomyces cerevisiae using response surface methodology. Annual research and review in biology.

[24] Liu HZ, Wang Q, Liu YY, Fang F (2009). Statistical optimization of culture media and conditions for production of mannan by Saccharomyces cerevisiae. Biotechnology and bioprocess engineering.

[25] Dikit P, Maneerat S, H‐kittikun A (2012). Mannoprotein from spent yeast obtained from thai traditional liquor distillation: extraction and characterization. Journal of food process engineering.

[26] Galinari É, Sabry DA, Sassaki GL, Macedo GR, Passos FML, Mantovani HC, Rocha HAO (2017). Chemical structure, antiproliferative and antioxidant activities of a cell wall α-d-mannan from yeast Kluyveromyces marxianus. Carbohydrate polymers.

[27] Huang G, Yang Q, Wang ZB (2010). Extraction and deproteinization of mannan oligosaccharides. Zeitschrift für naturforschung C journal of biosciences.

[28] Liu HZ, Liu L, Hui H, Wang Q (2015). Structural characterization and antineoplastic activity of Saccharomyces cerevisiae mannoprotein. International journal of food properties.

[29] Waterborg JH, Matthews HR (1994). The Lowry method for protein quantification. Methods in molecular biology.

[30] Kavanagh F (1981). Official Methods of Analysis of the AOAC 13th ed. Journal of pharmaceutical sciences.

[31] Liu XY, Wang Q, Cui SW, Liu HZ (2008). A new isolation method of β-D-glucans from spent yeast Saccharomyces cerevisiae. Food hydrocolloids.

[32] Levitz SM, Huang H, Ostroff GR, Specht CA (2015). Exploiting fungal cell wall components in vaccines. Seminars in immunopathology.

[33] Colm AM (2004). Functional Components of the cell wall of Saccharomyces cerevisiae: applications for yeast glucan and mannan. International Feed Industry Symposium.

[34] Duan S, Huang Q, Shen X, Hu J, Yi X, Li Z, Ding B (2020). Deproteinization of four macroporous resins for rapeseed meal polysaccharides. Food science and nutrition.

[35] Huang G, Shu S, Cai T, Liu Y, Xiao F (2012). Preparation and deproteinization of garlic polysaccharide. International research on food science and nutrition.

[36] Huang G, Chen Y, Wang X (2011). Extraction and deproteinization of pumpkin polysaccharide. International research on food science and nutrition.

[37] Huang GL, Yang Q, Wang ZB (2010). Extraction and deproteinization of mannan oligosaccharides. Zeitschrift für Naturforschung C journal of biosciences.

